# Non-high-density lipoprotein cholesterol predicts cardiovascular risk better than remnant cholesterol in patients with type 2 diabetes mellitus

**DOI:** 10.3389/fcvm.2025.1551203

**Published:** 2025-06-30

**Authors:** Paul Nsiah, Samuel Acquah, Ansumana Sandy Bockarie, George Adjei, Ebenezer Aniakwaa-Bonsu, Oksana Ryabinina

**Affiliations:** ^1^ Department of Chemical Pathology, School of Medical Sciences, College of Health and Allied Sciences, University of Cape Coast, Cape Coast, Ghana; ^2^ Department of Medical Biochemistry, School of Medical Sciences, College of Health and Allied Sciences, University of Cape Coast, Cape Coast, Ghana; ^3^ Department of Internal Medicine and Therapeutics, School of Medical Sciences, College of Health and Allied Sciences, University of Cape Coast, Cape Coast, Ghana; ^4^ Department of Community Medicine, School of Medical Sciences, College of Health and Allied Sciences, University of Cape Coast, Cape Coast, Ghana; ^5^ Department of Microbiology and Immunology, School of Medical Sciences, College of Health and Allied Sciences, University of Cape Coast, Cape Coast, Ghana

**Keywords:** non-high-density lipoprotein cholesterol, remnant cholesterol, type 2 diabetes mellitus, cardiovascular risk, insulin resistance, atherogenic dyslipidemia, metabolic syndrome, predictive biomarkers

## Abstract

**Background:**

Cardiovascular disease (CVD) is a leading cause of morbidity in patients with type 2 diabetes mellitus (T2DM). Non-high-density lipoprotein cholesterol (non-HDL-c) and remnant cholesterol (RC) have emerged as promising markers of atherogenic risk, but their comparative predictive performance remains uncertain, particularly in resource-limited settings.

**Objective:**

This study evaluated the predictive value of non-HDL-c and RC for atherosclerotic cardiovascular disease (ASCVD) risk and associated inflammatory and metabolic disturbances in T2DM patients.

**Methods:**

A cross-sectional study was conducted among 154 T2DM patients attending the outpatient diabetic clinic at the Effia Nkwanta Regional Hospital, Ghana. Non-HDL-c and RC were calculated from fasting lipid profiles. The TyG index was used as a surrogate for insulin resistance. ASCVD risk was assessed using the Framingham risk score. Logistic regression and ROC analysis were performed to assess predictive utility. Subgroup analyses were conducted based on BMI, hypertension, and TyG index.

**Results:**

Non-HDL-C was significantly associated with higher ASCVD risk and elevated hs-CRP and resistin levels, while RC showed weaker, non-significant associations. Non-HDL-c had a higher AUC (0.78 vs. 0.62), sensitivity, and specificity. Nearly half of participants (49.4%) had elevated TyG index (>8.7). Non-HDL-C consistently outperformed RC across subgroups.

**Conclusion:**

Non-HDL-c is a stronger and more practical predictor of ASCVD risk than RC in T2DM patients, particularly in settings with limited access to advanced lipid testing. Its use alongside the TyG index offers a cost-effective approach for enhancing cardiovascular risk stratification in diabetes care.

## 1 Introduction

It is well known that people with diabetes have an elevated atherossclerotic cardiovascular disease (ASCVD) risk. Patients with diabetes have a two to four times higher risk of experiencing cardiovascular events than those without diabetes (1), and their relative risk of dying from ASCVD is about twice as high (2). Growing evidence suggests that dyslipidaemia contributes significantly to the excess risk of ASCVD (3).

Common characteristic features of diabetic dyslipidaemia are the elevation of plasma triglycerides and triglyceride-rich very low-density lipoprotein (VLDL) cholesterol, reduced high-density lipoprotein cholesterol (HDL-c), and an increased number of small dense low-density lipoprotein cholesterol (LDL-c) particles (4) Although LDL-c is not typically elevated in patients with diabetes, the changes in LDL-c composition that can accompany the disease make the LDL-c exceptionally atherogenic (5). Traditionally, patients are evaluated for dyslipidaemia with respect to lipids of total cholesterol (TC), LDL-c, HDL-c, and triglycerides with attention to LDL-c values (6). According to the National Cholesterol Education Program (NCEP), LDL-c is still considered the primary target of lipid-lowering therapy for ASCVDs (7, 8) the sum of all lipoproteins that have atherogenic properties (7).

Remnant cholesterol is the cholesterol content of triglyceride-rich lipoproteins (TRLs) that consists of VLDL, intermediate-density lipoproteins (IDL), and chylomicron remnants (9) Epidemiological evidence has suggested that higher RC levels are significantly associated with the development of type 2 diabetes mellitus (T2DM) and contribute to comorbidities such as hypertension (10). RC is calculated from a standard lipid profile as total cholesterol minus low-density lipoprotein (LDL) cholesterol minus high-density lipoprotein (HDL) cholesterol. A recent study on ASCVD risk assessment indicated that current high-density lipoprotein cholesterol–based risk calculations could lead to inaccurate risk assessment in Black adults (11). (This raises a lot of issues about the estimation of cardiovascular disease risk scores).

Non-high-density lipoprotein cholesterol (non-HDL-c) encompasses all atherogenic apolipoprotein B–containing lipoproteins, including LDL, VLDL, IDL, and remnant particles. Unlike LDL-C, which primarily reflects cholesterol carried by low-density lipoproteins, non-HDL-C provides a more comprehensive measure of atherogenic burden (12). Unlike LDL-C, which primarily reflects cholesterol carried by low-density lipoproteins, non-HDL-C provides a more comprehensive measure of atherogenic burden (13). Several studies have demonstrated its superior predictive value for atherosclerotic cardiovascular disease (ASCVD), particularly in individuals with metabolic disturbances such as diabetes mellitus (14, 15). Non-HDL-C, calculated as total cholesterol minus HDL-C, represents the cholesterol content of all atherogenic lipoproteins including LDL, VLDL, IDL, lipoprotein(a), and remnant lipoproteins. In contrast, remnant cholesterol (RC) refers specifically to the cholesterol content of triglyceride-rich lipoproteins such as VLDL and chylomicron remnants and is usually estimated by subtracting LDL-C and HDL-C from total cholesterol. While both markers reflect atherogenic burden, non-HDL-C offers broader lipoprotein coverage and is endorsed by clinical guidelines as a secondary therapeutic target, especially in individuals with elevated triglycerides or diabetes (15).

Despite evidence from other regions suggesting the utility of non-HDL-c and RC in predicting ASCVD risk, their applicability and superiority have not been extensively studied in sub-Saharan African populations with T2DM [32; 10; (16)]. Obesity plays a critical role in the pathogenesis of T2DM and its associated cardiovascular complications. It is strongly linked to insulin resistance, chronic low-grade inflammation, and the dysregulation of adipokines such as adiponectin and resistin, which in turn contribute to atherogenic dyslipidaemia (17). Adiponectin, resistin, and high-sensitivity C-reactive protein (hs-CRP) are key biomarkers that reflect inflammatory and metabolic disturbances in T2DM. Adiponectin is an anti-inflammatory adipokine that is typically reduced in individuals with obesity and insulin resistance; levels below 5.0 µg/ml are often considered low and associated with increased cardiovascular risk. Resistin, a pro-inflammatory cytokine, is secreted by adipose tissue and monocytes, and elevated levels (>10.0 ng/ml) have been linked to insulin resistance, endothelial dysfunction, and atherosclerosis. hs-CRP, an established marker of subclinical inflammation, is considered elevated at levels >3.0 mg/dl and is associated with a heightened risk of cardiovascular events (18–20). Obese individuals with T2DM often present with elevated plasma triglycerides, increased VLDL cholesterol, decreased HDL cholesterol, and an excess of small dense LDL particles—collectively known as diabetic dyslipidaemia (21). These lipid abnormalities are captured more comprehensively by non-HDL-C and RC, making these markers clinically relevant in the assessment of cardiovascular risk in overweight and obese patients with diabetes. Therefore, understanding the contribution of obesity to lipid and metabolic disturbances provides important context for interpreting the predictive value of non-HDL-C and RC in this study. The understanding of the role of obesity as a risk factor is still debated (22). The concept of the obesity paradox, which was first observed in coronary artery disease patients, hypothesizes that some individuals with normal weight could have worse outcomes than their overweight/obese counterparts (23). The role of non-routine lipid biomarkers in the obesity paradox has not been widely explored. Insulin resistance is a hallmark of T2DM and plays a central role in the development of dyslipidaemia and atherosclerosis. It contributes to increased hepatic VLDL production, reduced lipoprotein lipase activity, and impaired HDL metabolism, all of which elevate levels of non-HDL-C and remnant cholesterol (24). Resistin, an adipocytokine secreted by macrophages and adipose tissue, has been implicated in insulin resistance, endothelial dysfunction, and chronic inflammation—key drivers of cardiovascular disease (25). Elevated resistin levels have been positively associated with atherogenic lipoproteins, including non-HDL-C and RC. Furthermore, metabolic syndrome—a cluster of risk factors including abdominal obesity, hypertension, dyslipidaemia, and impaired glucose metabolism—is highly prevalent in T2DM and significantly increases ASCVD risk (26). Both non-HDL-C and RC are recognized as lipid markers strongly linked to the presence and severity of metabolic syndrome (27). Including these variables in our analysis provides a more integrated evaluation of residual cardiovascular risk in this population. Therefore, the current study was designed to compare the respective predictive values of LDL-c, non-HDL-c, and RC for ASCVD in diabetes patients.

This study was guided by the hypothesis that non-HDL cholesterol, as a composite marker of atherogenic lipoproteins, is a better predictor of cardiovascular disease (CVD) risk among T2DM patients than remnant cholesterol (RC). We further hypothesized that a defined cut-off of non-HDL-c would independently predict elevated ASCVD risk in this population*.*

## 2 Materials and methods

### 2.1 Study design, population and sampling

A cross-sectional study was conducted at the diabetic clinic of Effia Nkwanta Regional Hospital, Takoradi, Ghana. The Effia Nkwanta Regional Hospital is a hub for referrals to outlying healthcare facilities in the Western Region. Patients with diabetes can receive both general and specialty care at the diabetic clinic. Participants were selected using simple random sampling. A sampling frame of all eligible type 2 diabetes patients attending the diabetes clinic was created from the clinic registry. To minimize selection bias, we used simple random sampling. A list of all eligible patients meeting the inclusion criteria was compiled, and computer-generated random numbers in Microsoft Excel were used to randomly select 154 participants. This ensured that each patient had an equal and independent probability of being included in the study. The sample size was based on the number of patients available during the study period and logistical feasibility. Although no formal *a priori* power calculation was conducted, the sample was adequate for exploratory analysis and yielded statistically significant results in multivariable regression and ROC analysis.

### 2.2 Inclusion and exclusion criteria

Type 2 diabetes mellitus patients aged 40 years and older who visited the outpatient diabetes Clinic, as ASCVD risk significantly increases after the fourth decade of life. Also, standard ASCVD risk assessment models, such as the Framingham Risk Score, are validated for individuals aged 40 years and above. Only individuals who provided written informed consent were included in the study to ensure voluntary participation and adherence to ethical standards. Patients were excluded if they had:

• Hepatitis B or C infections, due to the impact of chronic liver disease on lipid profiles and inflammatory markers.
• Recent infections, which could transiently elevate inflammatory markers like hs-CRP and disrupt lipid metabolism, affecting cardiovascular risk assessment.
• Hormonal contraceptive use, as contraceptives can alter lipid parameters and coagulation profiles, introducing confounding effects.
• A history of alcoholism, because chronic alcohol consumption influences lipid metabolism, glycemic control, liver function, and systemic inflammation, thereby potentially biasing cardiovascular risk evaluation.

### 2.3 Blood pressure and anthropometric measurements

An experienced nurse at the diabetic clinic took the patient's blood pressure from the left upper arm using a mercury sphygmomanometer and a stethoscope. Prior to measurement, participants were instructed to rest for at least five minutes. The blood pressure reading was calculated as the average of two readings taken five minutes apart. Hypertension was defined as systolic BP ≥140 mmHg or diastolic BP ≥90 mmHg, or current use of antihypertensive medication, according to the 2017 ACC/AHA guideline (28).

Height and weight were measured for the computation of body mass index (BMI). Height was measured barefoot using a wall-mounted ruler to the nearest 0.1 cm. Weight was measured in light clothing to the nearest 0.1 kg on a weighing scale. BMI was computed by dividing the weight by the square of the height, BMI = BMI (kg/m^2^) = weight/height^2^. Waist and hip circumferences were measured to the nearest 0.1 cm using a measuring tape. The waist was divided by the hip to obtain the waist-to-hip ratio (WHR). Total body fat (%) was estimated using the Omron BF511 Body Composition Monitor (Omron Corporation, Japan), as a complementary measure to BMI. This allowed for a more specific estimation of adiposity, as BMI does not differentiate between fat and lean mass. Including body fat percentage supported a more comprehensive evaluation of cardiometabolic risk factors among patients with type 2 diabetes mellitus.

### 2.4 Sample collection, preparation and biochemical assays

Five milliliters (5 ml) of venous blood sample was collected after an overnight fast of at least 8 h. One (1 ml) milliliter of blood was dispensed into tubes containing fluoride oxalate, and the remaining 4 ml of blood was dispensed into gel separator tubes. The sample of fluoride oxalate was used for fasting plasma glucose (FPG) estimation. The gel separator tubes were placed in a centrifuge and spun at 3,000 rpm for 5 min to obtain the serum. FPG was measured immediately using enzymatic colorimetric test kit, (GOD-PAP method) from Human Diagnostics Worldwide, Germany, and the serum for the measurement of other biochemical variables was aliquoted and stored at −20°C until analysis. Total cholesterol (TC), HDL-c, triglyceride (TG), LDL-c, FPG, alanine transaminase (ALT), and gamma-glutamyl transferase (GGT) were estimated using an automated chemistry analyzer (Selectra Pro S System, Elitech Group, France). Serum adiponectin, resistin, and high-sensitivity C-reactive protein (hs-CRP) were determined by commercially available enzyme-linked immunosorbent assay (ELISA) test kits procured from DRG International, following the manufacturer's instructions (DRG International, NJ, USA). Based on literature and the distribution of the study sample, the following cut-off values were used to define abnormal biomarker levels: hs-CRP >3.0 mg/dl (elevated inflammation), adiponectin <5.0 µg/ml (reduced anti-inflammatory adipokine), and resistin >10.0 ng/ml (increased pro-inflammatory state). These thresholds were used to analyze the associations between inflammatory/metabolic dysregulation and lipid markers such as non-HDL-c and RC.

The 10-year ASCVD risk for each participant was assessed using the Framingham Risk Score (FRS), based on the algorithm developed by D'Agostino et al. (29). A 10-year ASCVD risk score ≥20% was classified as high cardiovascular risk. Although ASCVD risk calculators may underestimate risk in Black populations, they remain widely used and endorsed by clinical guidelines. Their use in this study allows for comparability with other published works and enables the assessment of how non-HDL-c and RC correlate with an established risk prediction model, despite its known limitations. Insulin resistance was assessed by the triglyceride glucose index derived by the formula TyG = ln[FBS(mg/dl) × TG (mg/dl)]/two developed by Simental-Mendía et al. (30). A TyG index >8.7 was considered indicative of insulin resistance, based on previously published studies validating this cut-off in populations with type 2 diabetes mellitus.

## 3 Ethical consideration

The study was approved by the Institutional Review Board of University of Cape Coast (ID: UCCIRB/CHAS/2021/124). In addition, institutional approval was obtained from the Effia Nkwanta Regional Hospital. Furthermore, the conduct of the study was in strict adherence to the ethical standards of the Ghana Health Service (GHS) and the World Medical Association Declaration of Helsinki. Above all, written informed consent was obtained from each study participant, and strict confidentiality of participants’ information was maintained throughout the study.

## 4 Statistical analysis

Data obtained were analyzed by Statistical Package for Social Sciences (SPSS) software version 25. Data are presented as mean ± standard deviation (SD) or percentages, where appropriate. Independent sample *t*-test was used to compare the mean levels of measured indices between weight groups and between sexes. Pearson correlation, stepwise linear, and logistic regression analyses were performed. A *p*-value <0.05 was considered statistically significant. Multivariate logistic regression was used to determine adjusted odds ratios (AOR), controlling for potential confounders including age, sex, BMI, duration of diabetes, HbA1c, and hypertension.

## 5 Results

### 5.1 Clinical and biochemical characteristics of respondents

There were 154 participants aged 40–78 years and made up of 91 females and 63 males. The mean age was 52 years. Table 1 is a summary of the clinical and biochemical characteristics of the study participants. The fasting plasma glucose (FPG) level averaged 8.30 ± 4.70 mmol/L, indicating poor glycemic control, with levels ranging from 3.5 to 25.1 mmol/L.

**Table 1:** Clinical and biochemical characteristics of respondents.

Variable	Mean ± SD	Min–Max
Age (years)	52.20 ± 8.70	40–78
FPG (mmol/L)	8.30 ± 4.70	3.5–25.1
TC (mmol/L)	5.10 ± 1.00	3.1–7.8
TG (mmol/L)	1.20 ± 0.50	0.40–2.90
HDL-c (mmol/L)	1.20 ± 0.20	0.70–1.60
LDL-c (mmol/L)	3.10 ± 1.00	1.20–6.10
Non-HDL-c (mmol/L)	3.90 ± 1.00	1.60–6.90
RC (mmol/L)	0.54 ± 0.23	0.20–1.30
TyG index	8.70 ± 0.80	7.40–10.70
BMI (kg/m^2^)	26.90 ± 4.10	15.10–40.50
ASCVD risk (%)	14.30 ± 8.70	3.00–45.00
hs-CRP (mg/dl)	1.60 ± 0.80	0.11–3.80
Adiponectin (µg/ml)	7.70 ± 4.70	1.30–20.00
Resistin (ng/ml)	13.50 ± 3.70	4.80–22.30
Systolic BP (mmHg)	142 ± 16.00	102–197
Diastolic BP (mmHg)	88 ± 11.00	64–124
MAP (mmHg)	106 ± 12.00	78–146

FPG, fasting plasma glucose; HDL-c, high-density lipoprotein cholesterol; LDL-c low-density lipoprotein cholesterol; RC, remnant cholesterol; ASCVD, atherosclerotic cardiovascular disease; hs-CRP, high sensitivity C-reactive protein; BP, blood pressure; MAP, mean arterial pressure; BMI, body mass index; TG, triglycerides; WC, waist circumference; WHR, waist-to-hip ratio; ALT, alanine aminotransferase; GGT gamma-glutamyl transferase; TC, total cholesterol; TyG, triglyceride glucose index; Min, minimum; Max, maximum.

High-density lipoprotein cholesterol (HDL-c) was 1.20 ± 0.20 mmol/L, while low-density lipoprotein cholesterol (LDL-c) and non-HDL cholesterol (non-HDL-c) averaged 3.10 ± 1.00 mmol/L and 3.90 ± 1.00 mmol/L, respectively. Remnant cholesterol (RC) was relatively low, with a mean of 0.54 ± 0.23 mmol/L. The TyG index, an indicator of insulin resistance, had a mean of 8.70 ± 0.80, suggesting a high prevalence of insulin resistance among participants. Using the literature-established cut-off of >8.7 for the TyG index, calculated using glucose and triglycerides in mg/dl, 76 out of 154 participants (49.4%) were classified as having elevated insulin resistance Many participants were overweight or obese.

### 5.2 Clinical and biochemical characteristics of respondents by weight category

Respondents were grouped into BMI <25 kg/m^2^ and BMI >25 kg/m^2^ categories for comparison of the various indices (Table 2). Total cholesterol, LDL, non-HDL cholesterol, blood pressure, hs-CRP, resistin, and fat percentage levels were significantly higher in the BMI ≥25 kg/m^2^ group. Adiponectin level was significantly lower in the BMI ≥25 kg/m^2^ group, with the remaining indices being comparable between the two weight groups.

**Table 2:** Clinical and biochemical characteristics of respondents by weight category.

Parameter	BMI < 25 kg/m^2^ (40)	BMI ≥ 25 kg/m^2^ (114)	*P*-value
Age (years)	53.80 ± 10.13	53.09 ± 8.09	0.655
Duration (years)	4.04 ± 3.49	4.51 ± 3.09	0.482
FPG (mmol/L)	8.24 ± 4.75	8.26 ± 4.75	0.981
TC (mmol/L)	4.58 ± 0.74	5.22 ± 1.00	<0.001*
HDL-c (mmol/L)	1.19 ± 0.22	1.2 ± 0.20	0.774
LDL-c (mmol/L)	2.85 ± 0.78	3.47 ± 0.74	<0.001*
TyG Index	8.72 ± 0.76	8.76 ± 0.76	0.817
TG (mmol/L)	1.18 ± 0.59	1.19 ± 0.48	0.933
RC (mmol/L)	0.54 ± 0.27	0.54 ± 0.21	0.833
Non-HDL-c (mmol/L)	3.39 ± 0.81	4.02 ± 1.06	0.001*
ASCVD Risk (%)	13.05 ± 7.96	14.78 ± 8.90	0.279
Diastolic BP (mmHg)	84.78 ± 9.93	88.93 ± 10.68	0.033*
Systolic BP (mmHg)	137.63 ± 15.93	143.45 ± 16.65	0.047*
ALT (U/L)	22.80 ± 6.87	23.81 ± 7.42	0.451
GGT (U/L)	22.3 ± 7.10	22.68 ± 7.85	0.617
Adiponectin (µg/ml)	9.14 ± 4.83	7.15 ± 4.58	0.021*
Resistin (ng/ml)	11.56 ± 3.89	14.20 ± 3.66	<0.001*
hs-CRP	1.27 ± 0.70	1.72 ± 0.87	0.004*
Fat (%)	23.58 ± 7.47	33.04 ± 8.03	<0.001*

FPG, fasting plasma glucose; HDL-c, high-density lipoprotein cholesterol; LDL-c, low- density lipoprotein cholesterol; RC, remnant cholesterol; ASCVD, atherosclerotic cardiovascular disease; hs-CRP, high sensitivity C-reactive protein; BP, blood pressure; BMI, body mass index; TC, total cholesterol.

*Significant *p*-value.

### 5.3 Comparison of clinical and biochemical characteristics of respondents by hypertension status

Since the majority (75%) of respondents were hypertensive on appropriate treatment regimens, various indices were compared between the hypertensive and normotensive groups regardless of gender and weight category to examine the effects of hypertension on the measured indices. As expected, the hypertensive group demonstrated significantly (*P* < 0.05; Table 3) higher levels of all measured indices except adiponectin, which was lower than their normotensive counterparts with FPG, HDL-c and WHR being comparable (*P* > 0.05; Table 3) in the two groups.

**Table 3:** Comparison of clinical and biochemical characteristics of respondents by hypertension status.

Parameter	Normotensive (39)	Hypertensive (115)	*P*-value
FPG (mmol/L)	7.90 ± 5.00	8.40 ± 4.70	0.58
TC (mmol/L)	4.50 ± 0.10	5.20 ± 0.10	<0.001*
HDL-c (mmol/L)	1.20 ± 0.19	1.20 ± 0.21	0.29
TG (mmol/L)	1.00 ± 0.50	1.30 ± 0.50	0.004*
TyG index	8.50 ± 0.80	8.80 ± 0.70	0.02*
LDL-c (mmol/L)	2.90 ± 0.80	3.50 ± 1.00	0.001*
RC (mmol/L)	0.50 ± 0.20	0.60 ± 0.20	0.004*
Non-HDL-c (mmol/L)	3.30 ± 0.10	4.00 ± 1.00	0.001*
ASCVD risk (%)	8.30 ± 5.80	16.40 ± 8.50	<0.001*
hs-CRP (mg/dl)	1.00 ± 0.57	1.80 ± 0.80	<0.001*
Resistin (ng/ml)	11.30 ± 2.80	14.30 ± 3.60	<0.001*
Adiponectin (µg/ml)	11.40 ± 4.00	6.40 ± 4.20	<0.001*
BMI (kg/m^2^)	24.60 ± 3.45	27.60 ± 4.10	<0.001*
WHR	0.86 ± 0.06	0.89 ± 0.05	0.416
WC (cm)	87.40 ± 10.40	92.90 ± 8.40	0.001*
ALT (U/L)	22.20 ± 6.80	24.00 ± 7.40	0.17
GGT (U/L)	23.20 ± 8.20	22.30 ± 7.50	0.55
Duration (years)	2.80 ± 2.30	4.90 ± 3.30	0.002*
Fat (%)	24.43 ± 6.78	32.66 ± 8.58	<0.001

FPG, fasting plasma glucose; HDL-c, high-density lipoprotein cholesterol; LDL-c, low-density lipoprotein cholesterol; RC, remnant cholesterol; ASCVD, atherosclerotic cardiovascular disease; hs-CRP, high sensitivity C-reactive protein; BP, blood pressure; MAP, mean arterial pressure; BMI, body mass index; TG, triglycerides; WC, waist circumference; WHR, waist-to-hip ratio; ALT, alanine aminotransferase; GGT, gamma-glutamyl transferase; TyG, triglyceride glucose index; TC, total cholesterol.

*Significant *p*-value.

### 5.4 Association between non-HDL cholesterol, remnant cholesterol, and inflammatory and metabolic biomarkers

Table 4 presents the multiple linear regression analysis examining the associations between RC, non-HDL-c, and key inflammatory/metabolic biomarkers among T2DM patients. For non-HDL-c, significant inverse associations were observed with hs-CRP (*β* = −2.41, 95% CI: −3.98 to −0.85, *p* = 0.003) and adiponectin (*β* = −0.72, 95% CI: −1.21 to −0.23, *p* = 0.005), and a positive association was found with resistin (*β* = 1.08, 95% CI: 0.41–1.75, *p* = 0.002). In contrast, RC showed no significant associations with hs-CRP (*β* = −0.85, *p* = 0.278), adiponectin (*β* = −0.21, *p* = 0.412), or resistin (*β* = 0.36, *p* = 0.345). Neither RC nor non-HDL-c was significantly associated with the TyG index. These results suggest that non-HDL-c is a stronger predictor of inflammatory and metabolic disturbances than RC in this population of patients with diabetes.

**Table 4:** Linear regression analysis showing association between remnant cholesterol (RC), non-HDL cholesterol (non-HDL-c), and inflammatory/metabolic biomarkers among T2DM patients.

Predictor	Outcome variable	β coefficient (95% CI)	*p*-value
RC	hs-CRP	−0.85 (−2.40, 0.70)	0.278
Non-HDL-c	hs-CRP	−2.41 (−3.98, −0.85)	0.003*
RC	TyG Index	0.42 (−1.90, 2.74)	0.722
Non-HDL-c	TyG Index	1.37 (−2.32, 5.06)	0.464
RC	Adiponectin	−0.21 (−0.72, 0.30)	0.412
Non-HDL-c	Adiponectin	−0.72 (−1.21, −0.23)	0.005*
RC	Resistin	0.36 (−0.40, 1.12)	0.345
Non-HDL-c	Resistin	1.08 (0.41, 1.75)	0.002*

Results from multiple linear regression models, adjusted for age, sex, BMI, hypertension status, HbA1c, and duration of diabetes. β coefficients represent the estimated change in the outcome variable per unit increase in the predictor; RC, remnant cholesterol; hs-CRP, high sensitivity C-reactive protein; TyG, triglyceride glucose index.

* Significant *p*-value.

### 5.5 Associations between lipid markers and CVD risk

The association between remnant cholesterol (RC) and non-HDL cholesterol with atherosclerotic cardiovascular disease (ASCVD) risk, expressed as crude odds ratios (COR) and adjusted odds ratios (AOR) shown in Table 5. Abnormal RC levels were associated with a crude odds ratio (COR) of 1.70 (95% CI: 0.64–4.53) for ASCVD risk. After adjusting for confounders, the adjusted odds ratio (AOR) was 1.39 (95% CI: 0.50–3.85). Participants with abnormal non-HDL-c levels had a significantly higher likelihood of ASCVD risk. The crude odds ratio and the adjusted odds ratio (AOR) remained strong and significant.

**Table 5:** Association of remnant cholesterol (RC) and Non-HDL cholesterol with atherosclerotic cardiovascular (ASCVD) risk.

Variable	ASCVD risk no (*n*, %)	ASCVD risk yes (*n*, %)	COR (95% CI)	AOR (95% CI)
RC normal	39 (86.67%)	86 (79.63%)	1 (ref.)	1 (ref.)
RC abnormal	6 (13.33%)	22 (20.37%)	1.70 (0.64–4.53)	1.39 (0.50–3.85)
Non-HDL-c normal	26 (56.52%)	28 (25.93%)	1 (ref.)	1 (ref.)
Non-HDL-c abnormal	20 (43.48%)	80 (74.07%)	3.71 (1.80–7.67)*	3.60 (1.73–7.47)*

COR, crude odds ratio; AOR, adjusted odds ratio. Adjusted models include age, sex, and duration of diabetes. Statistically significant results (*p* < 0.05) are marked with an asterisk (*); ASCVD, atherosclerotic cardiovascular disease; RC, remnant; Non-HDL-c, non-HDL cholesterol.

**Figure 1:**
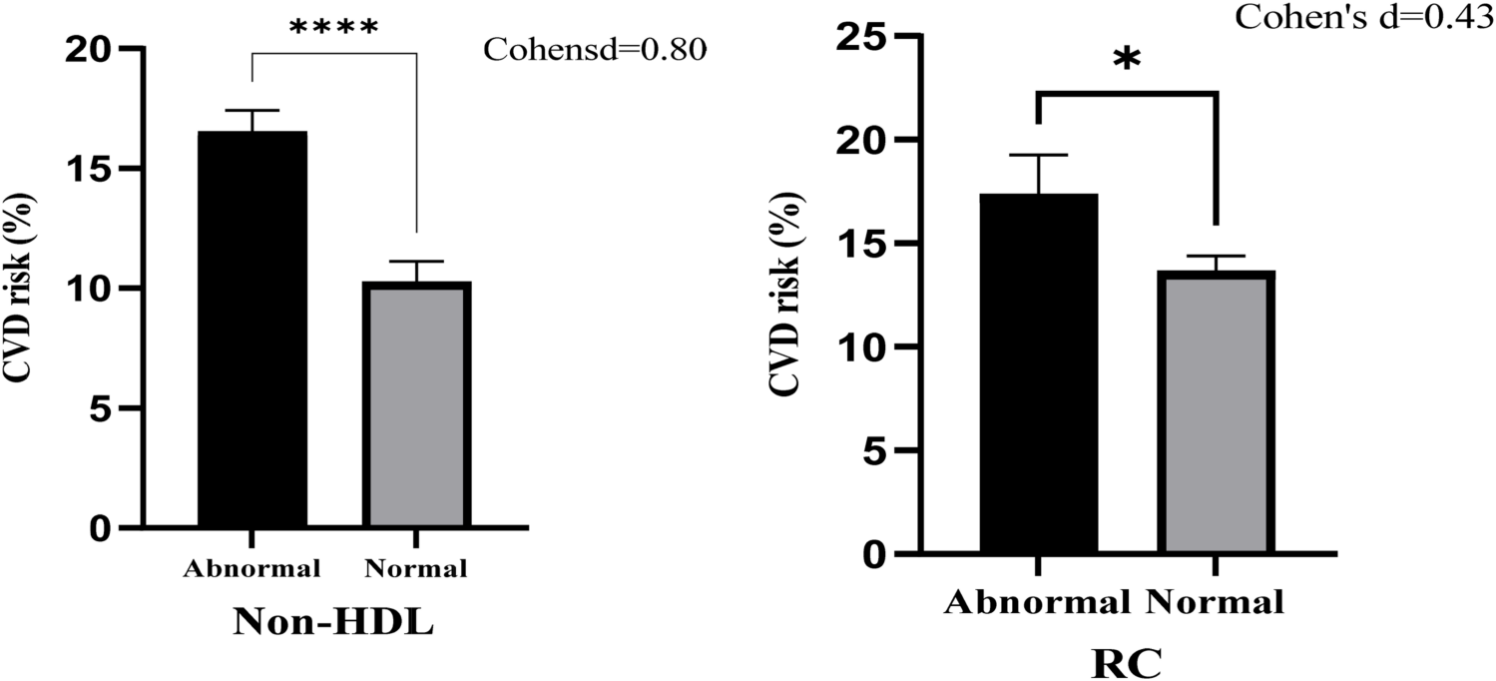
Bar chart with error bars of ASCVD risk associated with Non-HDL cholesterol and remnant cholesterol (RC).

The relationship between ASCVD risk and cholesterol measures [non-HDL cholesterol and remnant cholesterol (RC)] stratified by normal and abnormal levels is represented in Figure 1. Participants with abnormal non-HDL-c levels exhibited a significantly higher mean ASCVD risk percentage compared to those with normal levels. The mean ASCVD risk for abnormal non-HDL-c was approximately 17%, while it was around 11% for normal levels. This difference was statistically significant (****, *p* < 0.0001), with a large effect size (Cohen's *d* = 0.80). Participants with abnormal RC levels also had a higher mean ASCVD risk percentage compared to those with normal RC levels. The mean ASCVD risk was approximately 16% for abnormal RC and 13% for normal RC levels. This difference was statistically significant (*, *p* < 0.05), but the effect size was moderate (Cohen's *d* = 0.43).

### 5.6 Association of remnant cholesterol (RC) and non-HDL cholesterol with various biomarkers and metabolic syndrome

The association of RC and non-HDL-c with key biomarkers, including hs-CRP, TyG index, resistin, adiponectin, and metabolic syndrome, expressed as crude odds ratios (COR) and adjusted odds ratios (AOR), is depicted in Table 6. Abnormal RC levels were associated with an increased CRP level in the unadjusted model. However, this association was not significant in the adjusted model.

**Table 6:** Association of remnant cholesterol (RC) and non-HDL cholesterol with various biomarkers and metabolic syndrome.

Variable	No (*n*, %)	Yes (*n*, %)	COR (95% CI)	AOR (95% CI)
hs-CRP				
RC normal	44 (75.86%)	82 (85.42%)	1 (ref.)	1 (ref.)
RC abnormal	14 (24.14%)	14 (14.58%)	1.86 (0.82–4.23)	1.12 (0.34–3.65)
Non-HDL-c normal	10 (17.24%)	44 (45.83%)	1 (ref.)	1 (ref.)
Non-HDL-c abnormal	48 (82.76%)	52 (54.17%)	0.25 (0.11–0.54)*	0.31 (0.13–0.74)*
TyG index				
RC normal	28 (100.00%)	98 (77.78%)	1 (ref.)	1 (ref.)
RC abnormal	0	28 (22.22%)	1	1
Non-HDL-c normal	18 (64.29%)	36 (28.57%)	1 (ref.)	1 (ref.)
Non-HDL-c abnormal	10 (35.71%)	90 (71.43%)	4.51 (1.90–10.68)*	2.69 (0.91–7.94)
Resistin				
RC normal	53 (92.98%)	73 (75.26%)	1 (ref.)	1 (ref.)
RC abnormal	4 (7.02%)	24 (24.74%)	4.36 (1.43–13.30)*	4.27 (0.93–19.65)
Non-HDL-c normal	33 (57.89%)	21 (21.65%)	1 (ref.)	1 (ref.)
Non-HDL-c abnormal	24 (42.11%)	76 (78.35%)	4.98 (2.43–10.16)*	5.24 (2.20–12.50)*
Adiponectin				
RC normal	74 (80.43%)	52 (85.25%)	1 (ref.)	1 (ref.)
RC abnormal	18 (19.57%)	9 (14.75%)	0.71 (0.30–1.70)	0.78 (0.32–1.91)
Non-HDL-c normal	27 (29.35%)	27 (44.26%)	1 (ref.)	1 (ref.)
Non-HDL-c abnormal	65 (70.65%)	34 (55.74%)	0.52 (0.27–1.03)	0.54 (0.27–1.06)
Metabolic syndrome				
RC normal	71 (79.78%)	55 (84.62%)	1 (ref.)	1 (ref.)
RC abnormal	18 (20.22%)	10 (15.38%)	0.72 (0.31–1.68)	1.54 (0.43–5.52)
Non-HDL-c normal	34 (38.20%)	20 (30.77%)	1 (ref.)	1 (ref.)
Non-HDL-c abnormal	55 (61.80%)	45 (69.23%)	1.39 (0.71–2.74)	1.74 (0.72–4.06)

COR, crude odds ratio; AOR, adjusted odds ratio. Adjusted models include age, sex, and duration of diabetes. Statistically significant results (*p* < 0.05) are marked with an asterisk (*); ASCVD, Atherosclerotic Cardiovascular Disease; RC, remnant; Non-HDL-c, non-HDL cholesterol; hs-CRP, high sensitivity C-reactive protein; TyG, triglyceride glucose index.

Abnormal non-HDL-c levels were inversely associated with hs-CRP in both models, suggesting a potential protective role. Furthermore, abnormal non-HDL-c levels were significantly associated with the TyG index in both unadjusted and adjusted, indicating its link to insulin resistance.

Abnormal RC levels were strongly associated with elevated resistin levels. Non-HDL-c levels showed a significant association with increased resistin in both, reinforcing its link to inflammation and metabolic dysregulation.

Neither RC nor non-HDL-c showed a significant correlation with adiponectin levels in both models.

Abnormal RC levels showed no significant association with metabolic syndrome in either unadjusted or adjusted models. However, abnormal non-HDL-c levels showed a marginally increased association with metabolic syndrome.

### 5.7 Predictive value of non-HDL cholesterol and remnant cholesterol: ROC curve analysis

**Figure 2:**
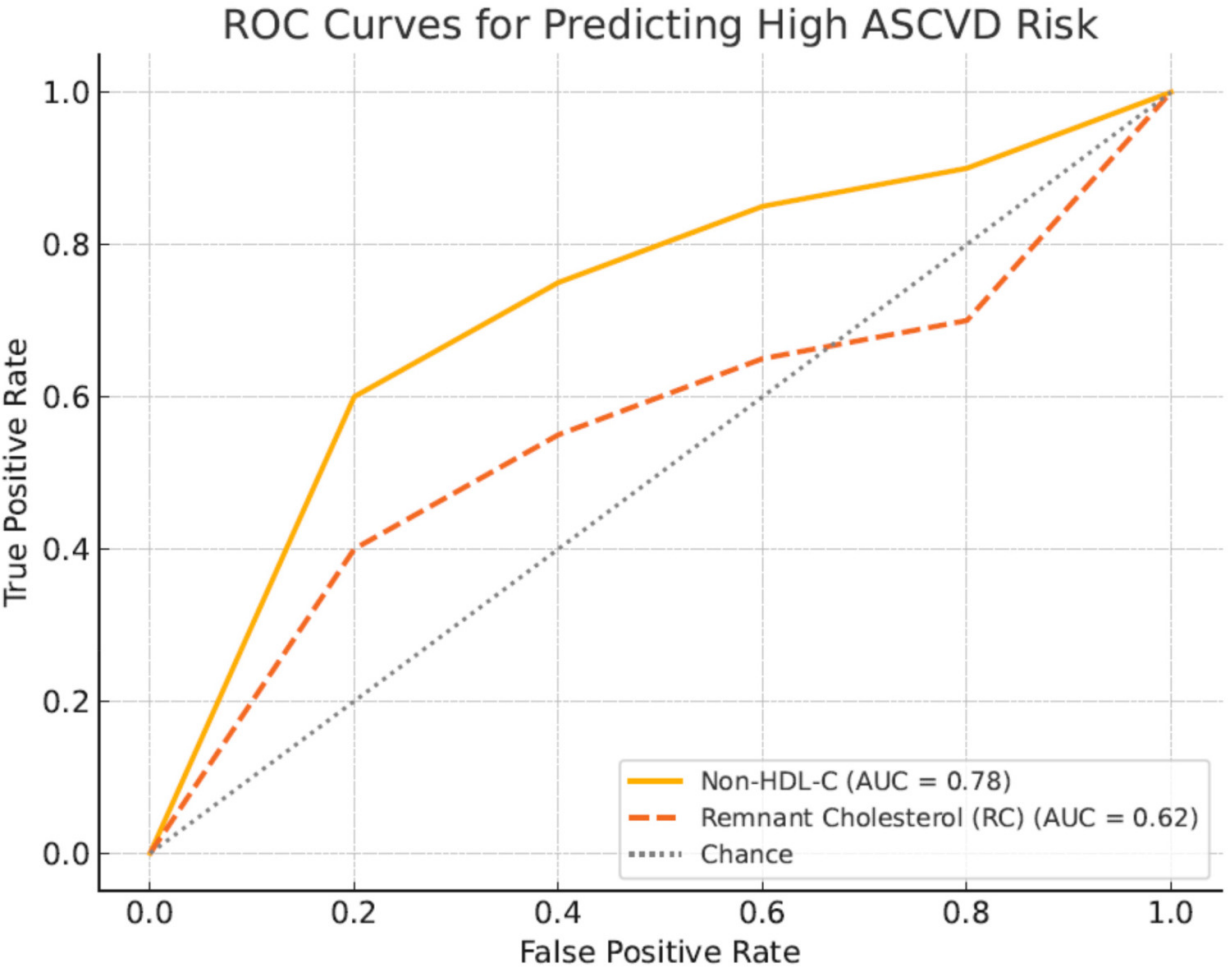
ROC curves comparing predictive ability of Non-HDL cholesterol and remnant cholesterol for ASCVD risk. Receiver operating characteristic (ROC) curves illustrate the comparative predictive performance of non-HDL-c (blue curve) and remnant cholesterol (red curve) for ASCVD risk in the study cohort. The AUC for non-HDL-c was 0.81, indicating strong predictive accuracy, whereas RC showed an AUC of 0.66.

To evaluate the predictive value of non-HDL-c and remnant cholesterol (RC) for ASCVD risk, receiver operating characteristic (ROC) curve analyses were conducted. The area under the curve (AUC) for non-HDL-c was 0.81 (95% CI: 0.74–0.88), indicating strong predictive ability, while RC showed a lower AUC of 0.66 (95% CI: 0.58–0.74), suggesting modest predictive power. The optimal cut-off point for non-HDL-c was identified at 3.7 mmol/L, yielding a sensitivity of 78% and specificity of 72%. For RC, the optimal cut-off was 0.9 mmol/L, with sensitivity of 65% and specificity of 60%. Figure 2 presents the ROC curves.

Table 7 presents the results of the receiver operating characteristic (ROC) analysis comparing the predictive ability of non-HDL cholesterol (non-HDL-c) and remnant cholesterol (RC) for ASCVD risk among T2DM patients. The area under the curve (AUC) for non-HDL-c was 0.81 (95% CI: 0.74–0.88), indicating strong predictive accuracy, while the AUC for RC was lower at 0.66 (95% CI: 0.58–0.74), reflecting moderate predictive performance. The optimal cut-off value for non-HDL-c was determined to be 3.7 mmol/L, yielding a sensitivity of 78% and specificity of 72%. For RC, the optimal cut-off point was 0.9 mmol/L, with a sensitivity of 65% and specificity of 60%. These findings demonstrate that non-HDL-c is a stronger discriminator of ASCVD risk in this cohort compared to RC, consistent with its broader atherogenic coverage.

**Table 7:** ROC analysis showing predictive performance of Non-HDL cholesterol and remnant cholesterol for ASCVD risk.

Predictor	AUC (95% CI)	Optimal cut-off (mmol/L)	Sensitivity (%)	Specificity (%)
Non-HDL-c	0.78 (0.71–0.85)	>4.1	81.1	56.5
Remnant cholesterol (RC)	0.62 (0.54–0.70)	>0.8	64.0	36.0

## 6 Discussion

### 6.1 Overview and glycemic control in the study population

This cross-sectional study in 154 type 2 diabetes mellitus (T2DM) patients at Effia-Nkwanta Regional Hospital examined the predictive power of remnant cholesterol (RC) and non-HDL cholesterol (non-HDL-c) for cardiovascular disease (CVD) risk prediction. The average age of participants was 52.20 ± 8.70 years (range: 40–78 years), representing a predominantly middle-aged to older demographic commonly affected by T2DM and its complications.

The mean fasting plasma glucose (FPG) level was 8.30 ± 4.70 mmol/L, reflecting poor glycemic control (Table 1). This is consistent with findings in other diabetes populations, such as in a study by Feleke et al. in Ethiopia, where mean FPG levels were also above optimal thresholds in sub-Saharan populations (16). According to the American Diabetes Association (ADA), the target FPG for adults with diabetes is 4.4–7.2 mmol/L (17, 18). Thus, addressing poor glycemic control is crucial to mitigate cardiovascular risk. Chronic hyperglycemia drives endothelial dysfunction, promotes oxidative stress, increases systemic inflammation, and exacerbates lipid abnormalities, all of which synergistically amplify ASCVD risk (31).

### 6.2 Obesity, lipid profile, and the obesity paradox

When the data were stratified by BMI, total cholesterol, LDL-c, non-HDL-c, blood pressure, hs-CRP, resistin, and percentage body fat were all higher among overweight and obese participants (BMI ≥25 kg/m^2^), whereas adiponectin levels were lower (Table 2). This pattern suggests that the “obesity paradox”—reported in conditions such as hypertension (19), T2DM (20), and COVID-19 (21) —was not evident in this study.

Higher TC, LDL-C, and non-HDL-c levels among the overweight/obese participants are consistent with dyslipidemia observed in poorly controlled T2DM patients (22). It is estimated that 30%–60% of T2DM patients have dyslipidemia (23). The 74% overweight/obesity prevalence in this study aligns with findings from a meta-analysis in Africa showing a prevalence range of 56.9%–88.5% (24). Moreover, elevated hs-CRP, resistin, and lower adiponectin levels in overweight participants indicate systemic inflammation and metabolic dysfunction, in line with previous observations (25).

### 6.3 Hypertension prevalence and its interactions with diabetes and lipids

Hypertension was present in 74.68% of participants (Table 3), a figure much higher than Ghana's general hypertension prevalence of 27% (26). This high prevalence is comparable to studies from Afghanistan (70.5%) (28) and Jordan (74.6%) (29) but higher than an Ethiopian report (37.5%) (27). Differences in age, type of diabetes, and obesity rates between study populations may explain these discrepancies.

Hypertension and diabetes share common pathophysiologic mechanisms such as inflammation and oxidative stress (30). Poor glycemic control (mean FPG = 8.3 mmol/L) likely contributed to hypertension development in this cohort, consistent with ADA recommendations of maintaining FPG between 4.0–7.5 mmol/L (31).

The linear regression analyses presented in Table 4 highlight important associations between non-HDL-c, RC), and key inflammatory and metabolic biomarkers among T2DM patients. Notably, non-HDL-c showed a significant positive association with resistin and an inverse association with hs-CRP, while RC showed no significant associations after adjustment. These findings suggest that non-HDL-c may play a more prominent role than RC in driving inflammatory and metabolic dysregulation in this population.

Our results align with the broader evidence linking non-HDL-c to cardiovascular risk pathways in diabetes. For example, Sattar et al. reported that younger age at T2DM diagnosis increases lifetime cardiovascular risk, in part due to prolonged exposure to adverse lipid profiles and inflammatory stress. Similarly (32), Ference et al. emphasized that non-HDL-c, which captures all atherogenic apoB-containing lipoproteins, is a superior predictor of cardiovascular events compared to LDL-c alone (33). The observed association between non-HDL-c and resistin in our study reinforces the role of non-HDL-c in promoting adipose tissue dysfunction, insulin resistance, and systemic inflammation.

In contrast, although RC has been shown to predict ischemic heart disease and myocardial infarction in general populations (34), our findings suggest that within T2DM patients, non-HDL-c may serve as a more comprehensive indicator of atherogenic burden. This is supported by current European guidelines, which prioritize non-HDL-c as a key therapeutic target, particularly in high-risk groups like T2DM (35).

Moreover, recent work on emerging lipid biomarkers such as lipoprotein(a) suggests that traditional markers like non-HDL-c remain central to cardiovascular risk assessment, even as newer targets are explored (36). Overall, our findings contribute to the growing recognition of non-HDL-c as both a practical and powerful marker for guiding lipid management strategies to reduce residual cardiovascular risk among patients with diabetes.

Although RC has been recognized for its potential causal contribution to atherosclerosis, primarily through endothelial dysfunction and inflammation, it is typically calculated as total cholesterol minus LDL-c and HDL-c, an approach that assumes accurate LDL-c estimation and is sensitive to triglyceride levels. This method may introduce variability, especially in diabetes patients with mixed dyslipidemia. In contrast, non-HDL-c is straightforward to compute from standard lipid profiles and includes all atherogenic lipoproteins, contributing to its superior performance in our study. This supports current guidelines that prioritize non-HDL-c as a practical and reliable cardiovascular risk marker (35).

### 6.4 Predictive value of remnant cholesterol versus non-HDL cholesterol

The findings show that RC did not significantly predict ASCVD risk after adjustment (AOR: 1.39; 95% CI: 0.50–3.85) (Table 5). This contrasts with studies by Nordestgaard et al., which identified RC as a strong predictor of ischemic heart disease (36). Differences may stem from the specific population studied—in T2DM, non-HDL-c may be a stronger predictor as it captures a broader range of atherogenic particles.

Non-HDL-c demonstrated strong, statistically significant associations with ASCVD risk, metabolic syndrome markers, and systemic inflammation. Participants with abnormal non-HDL-c levels were approximately 3.6 times more likely to exhibit metabolic syndrome features (AOR: 3.60; 95% CI: 1.73–7.47). This supports findings from Li et al. (37), who emphasized the importance of achieving non-HDL-c targets to reduce residual ASCVD risk.

Nonetheless, a meta-analysis indicated that non-HDL-c is not always superior to LDL-c for ASCVD prediction (38). Differences between studies could reflect population characteristics, lipid measurement techniques, follow-up durations, or the extent of statistical adjustments.

### 6.5 Comparative strength of non-HDL-c and RC in ASCVD risk prediction

Analysis of ASCVD risk using a bar chart (Figure 1) showed that participants with abnormal non-HDL-c had a higher mean ASCVD risk (∼17%) compared to normal non-HDL-c (∼11%), with a large effect size (Cohen's *d* = 0.80; *p* < 0.0001). Although abnormal RC levels also correlated with higher ASCVD risk (∼16% vs. ∼13%), the effect size was moderate (Cohen's *d* = 0.43; *p* < 0.05).

This suggests that while both markers are associated with ASCVD risk, non-HDL-c is a stronger and more consistent predictor—likely because it includes RC as part of its total. When stratified by patient subgroups, non-HDL-c consistently demonstrated stronger associations with ASCVD risk compared to RC. Among participants with BMI ≥25 kg/m^2^, non-HDL-c showed a higher odds ratio and area under the curve (AUC), suggesting its enhanced predictive power in obese individuals with elevated adiposity-linked inflammation. In hypertensive participants, non-HDL-c was also significantly associated with elevated hs-CRP and metabolic markers, whereas RC showed weaker and non-significant relationships. Similarly, in participants with elevated TyG index (>8.7), non-HDL-c had a stronger correlation with ASCVD risk scores, while RC failed to reach statistical significance. These subgroup findings reinforce the broader applicability of non-HDL-c as a robust atherogenic marker across metabolic phenotypes, particularly in patients with obesity, hypertension, or insulin resistance.

### 6.6 Associations of lipid markers with other biomarkers

Table 6 shows that abnormal RC levels had a non-significant association with hs-CRP. In contrast, abnormal non-HDL-c levels were significantly associated with lower hs-CRP (AOR: 0.31; 95% CI: 0.13–0.74), indicating a potential anti-inflammatory pattern when lipoproteins are well-controlled.

Non-HDL-c was also significantly associated with insulin resistance (TyG index) and elevated resistin levels, reinforcing its relevance in metabolic dysfunction. This is consistent with literature emphasizing non-HDL-c's utility in predicting residual cardiovascular risk (40). The TyG index was used as a surrogate marker for insulin resistance in this study due to its practicality and strong correlation with HOMA-IR. Although HOMA-IR is a well-established tool based on fasting insulin and glucose levels, its reliance on insulin assays limits its routine use, especially in resource-constrained environments. In contrast, the TyG index can be calculated using standard fasting glucose and triglyceride levels, making it more feasible and cost-effective. Studies have demonstrated that a TyG index cut-off value around 8.7 offers good diagnostic performance, with reported sensitivity and specificity exceeding 80% when compared with HOMA-IR for predicting insulin resistance (37).

The observed prevalence of elevated TyG index (>8.7) in our study population (49.4%) aligns well with previous studies conducted in similar populations. For instance, a 2023 study in *Cardiovascular Diabetology* reported a comparable prevalence of TyG >8.7 among adults at metabolic risk (38). Likewise, a 2014 study published in *Endocrinología y Nutrición* identified TyG cut-offs of 8.7 and 8.8 for women and men, respectively, in relation to insulin resistance (39). These findings support the external validity of our results and confirm the appropriateness of the >8.7 threshold for assessing insulin resistance risk in diverse populations.

Findings from Loh et al. (41) questioned the universal applicability of current non-HDL-c targets, suggesting remnant cholesterol could be a better marker in specific populations, such as Southeast Asians. Differences between these findings and ours could stem from ethnic and genetic variations, dietary habits, sample size differences, prevalence of comorbidities, healthcare access, and environmental factors.

### 6.7 Comparison of predictive performance based on ROC analysis

Our ROC analysis revealed that non-HDL-c demonstrated superior predictive performance for ASCVD risk compared to remnant cholesterol, with an AUC of 0.81 vs. 0.66, respectively. This finding aligns with prior studies emphasizing the broader atherogenic coverage of non-HDL-c, which includes cholesterol from LDL, VLDL, IDL, and remnant lipoproteins (33, 35) While RC has been identified as an independent risk factor for cardiovascular disease in general populations (34), its predictive strength appears diminished in T2DM patients, where non-HDL-c remains the more robust marker. These results reinforce current guideline recommendations that prioritize non-HDL-c as a therapeutic and monitoring target in high-risk populations, including individuals with diabetes.

### 6.8 Clinical implications for resource-limited settings

The findings of this study have practical implications for cardiovascular risk stratification in low-resource settings. Both non-HDL-c and the TyG index can be calculated from standard lipid and glucose panels, which are widely available and inexpensive (40). This contrasts with other atherogenic indices that require specialized assays (e.g., apoB, insulin). The superior predictive value of non-HDL-c across patient subgroups reinforces its suitability as a second-line target after LDL-C, especially in patients with hypertriglyceridemia or mixed dyslipidemia (41). Similarly, the TyG index offers a feasible surrogate for insulin resistance screening in settings where HOMA-IR or clamp studies are impractical (42). Integrating these measures into primary care protocols could improve early identification of high-risk patients and inform cost-effective interventions to reduce ASCVD burden among patients with T2DM.

### 6.9 Recommended clinical applications of non-HDL-c

Incorporating non-HDL-c as a secondary lipid target is supported by multiple international guidelines. The ESC/EAS 2019 guidelines recommend specific non-HDL-c targets based on cardiovascular risk categories, emphasizing its role in patients with elevated triglycerides or metabolic syndrome (43). Similarly, the 2018 AHA/ACC guidelines (44) and the National Lipid Association recognize non-HDL-c as a valuable secondary target, particularly in individuals with hypertriglyceridemia or insulin resistance (45). These recommendations underscore the clinical utility of non-HDL-c in comprehensive cardiovascular risk assessment and management.

## 7 Conclusion

In this study of patients with type 2 diabetes mellitus, non-HDL-c demonstrated stronger predictive value for ASCVD risk than RC. Non-HDL-c showed consistent associations with metabolic and inflammatory biomarkers, and outperformed RC across clinical subgroups. The TyG index identified nearly half of participants as insulin-resistant, supporting its utility in resource-limited settings. These findings highlight non-HDL-c and TyG index as practical, cost-effective markers for cardiovascular risk assessment in T2DM populations.

## 8 Limitation

One limitation of this study is the absence of a formal prior sample size calculation. While the sample was based on the number of eligible patients available during the study period, the statistically significant findings in multivariable and ROC analyses suggest that the sample was sufficient to detect meaningful associations. Nonetheless, future studies with larger and power-calculated samples are warranted to validate these findings.

Future large-scale, multi-ethnic studies are needed to validate the findings and refine lipid marker-based CVD risk stratification strategies.

## Data Availability

The raw data supporting the conclusions of this article will be made available by the authors, without undue reservation.
