# Supplementary material for: Non-high-density lipoprotein cholesterol predicts cardiovascular risk better than remnant cholesterol in patients with type 2 diabetes mellitus
Source: Front Cardiovasc Med. 2025 Jun 30;12:1551203. doi: 10.3389/fcvm.2025.1551203 (PMC12256453; doi:10.3389/fcvm.2025.1551203)
Supplement: Supplementary file 1 [file Datasheet1.pdf]

Supplementary Tables Using mmol/L Cut-Offs

Supplementary Table 1: Abnormal Parameters by BMI Group

Parameter (Cut-off)	BMI <25 (n/%)	BMI ≥25 (n/%)
FPG >7.0 mmol/L	18 (45.0%)	51 (44.7%)
TG >1.7 mmol/L	5 (12.5%)	18 (15.8%)
HDL-C <1.0 mmol/L	30 (75.0%)	84 (73.7%)
TC >5.2 mmol/L	8 (20.0%)	52 (45.6%)
LDL-C >3.4 mmol/L	10 (25.0%)	55 (48.2%)
hs-CRP >3.0 mg/L	2 (5.0%)	8 (7.0%)
Resistin >15 ng/mL	4 (10.0%)	55 (48.2%)
Adiponectin <5 μg/mL	28 (70.0%)	67 (58.8%)

Supplementary Table 2: Abnormal Parameters by Hypertension Status

Parameter (Cut-off)	Normotensive (n/%)	Hypertensive (n/%)
FPG >7.0 mmol/L	15 (38.5%)	27 (42.9%)
TG >1.7 mmol/L	3 (7.7%)	11 (17.5%)
HDL-C <1.0 mmol/L	32 (82.1%)	44 (69.8%)
TC >5.2 mmol/L	7 (17.9%)	27 (42.9%)
LDL-C >3.4 mmol/L	7 (17.9%)	29 (46.0%)
hs-CRP >3.0 mg/L	0 (0.0%)	8 (12.7%)
Resistin >15 ng/mL	3 (7.7%)	29 (46.0%)
Adiponectin <5 μg/mL	38 (97.4%)	27 (42.9%)
